# Editorial: Herpesviruses: pathogenesis and host interactions

**DOI:** 10.3389/fmicb.2026.1982352

**Published:** 2026-09-24

**Authors:** Elias A. Rahal, Hye-Ra Lee, Anna Luganini

**Affiliations:** 1 Department of Experimental Pathology, Immunology, and Microbiology, American University of Beirut, Beirut, Lebanon; 2 Center for Infectious Diseases Research (CIDR), American University of Beirut, Beirut, Lebanon; 3 Department of Biotechnology and Bioinformatics, College of Science and Technology, Korea University, Sejong, Republic of Korea; 4 Department of Lab Medicine, College of Medicine, Korea University, Seoul, Republic of Korea; 5 Department of Life Sciences and Systems Biology, University of Turin, Turin, Italy

**Keywords:** interferon signaling, nucleocytoplasmic trafficking, transcriptional reprogramming, viral latency and reactivation, virus-host interactions

Herpesviruses represent one of the most sophisticated models of virus-host co-evolution because of their ability to establish lifelong latent infections, modulate immune responses, and reactivate in response to changes in the cellular environment (Adler et al., 2017). Against the background of an already extensive literature and recent comprehensive collections on herpesvirus transmission, pathogenesis, and treatment, this Research Topic, entitled “*Herpesviruses: pathogenesis and host interactions*,” provides a distinctive cross-scale perspective by bringing together nine contributions that span subcellular host remodeling, metabolic adaptation, immune and clinical heterogeneity, viral detection, vaccine development, and bibliometric mapping.

Read together, the contributions gathered here address three interconnected questions: (i) how do herpesviruses reorganize intracellular trafficking, transcription, and metabolism to support replication? (ii) How are the magnitude and context of host immune responses associated with distinct clinical outcomes? (iii) How do emerging molecular and analytical approaches generate new hypotheses with different degrees of translational readiness? Table 1 provides, for each study, the virus (or broader topic) studied, the model used, the principal findings, the main limitations, and the translational implications. Importantly, the Research Topic encompasses narrative, experimental, clinical, observational, diagnostic, and bibliometric evidence; conclusions must therefore be calibrated to the inferential strength of each study design.

**Table 1:** Summary and critical appraisal of the contributions included in the Research Topic.

References	Virus/topic	Model or study design	Main finding	Main limitation	Translational implication
Bjerhem et al.	HSV-2	Comparative *ex vivo* analysis of PBMCs and plasma from 88 patients diagnosed with HSV-2 meningitis or genital herpes	Patients with a history of HSV-2 meningitis displayed greater spontaneous type I and III IFN production and stronger HSV-2 induced IFN-γ responses than patients with genital herpes	Cross-sectional sampling after the clinical episodes cannot establish whether the immune profile preceded or resulted from meningitis; the groups also differed in age. The study does not directly demonstrate trained immunity	Immune profiles may contribute to future biomarker or risk stratification approaches (prospective longitudinal confirmation is required)
Cao et al.	Multiple α-, β-, and γ-herpesviruses	Herpesvirus nucleocytoplasmic shuttling proteins	Viral shuttling proteins exploit nuclear transport pathways to regulate viral gene expression, replication, virion assembly, and immune evasion	Narrative review; evidence is heterogeneous across different viruses and proteins and no new experimental validation is provided	Viral-host nuclear transport interactions may represent antiviral targets (selectivity and therapeutic feasibility require experimental validation)
Chen et al.	PRV	Metabolic reprogramming and transcriptomic analysis in infected porcine cells	Enhanced nucleotide metabolism, amino acid pathways, and biosynthetic processes that support viral replication	The metabolomic analysis used a small sample size (*n* = 3) in a single non-neuronal cell line (PK 15), limiting its representation of metabolic changes in PRV's primary neuronal targets	Nucleotide metabolism identified as a potential target for antiviral intervention
Ge et al.	MDV; CIAV	Administration of MDV, used as a recombinant vaccine vector expressing VP1 and VP2 proteins of CIAV, to chickens	The vaccine elicited strong humoral and cellular immune responses while markedly reducing viral replication and disease severity	The use of specific pathogen free (SPF) chickens and a single CIAV strain limits the applicability of the findings to commercial poultry and diverse field conditions	This study highlights the promise of herpesvirus-based vectors as a strategy for controlling CIAV infection
Hartwick et al.	Multiple herpesviruses	Longitudinal molecular analysis of conjunctival samples from 18 patients with PCR-confirmed adenovirus-negative acute conjunctivitis, with follow-up for up to 3 weeks	HHV-7 DNA was detected in conjunctival samples from 4 of 18 patients, whereas HSV-1, HSV-2, and HHV-6 were not detected	The small sample size and detection of HHV-7 DNA alone do not establish a causal relationship between HHV-7 and conjunctivitis	HHV-7 may represent a potential non adenoviral etiology of conjunctivitis and warrants further investigation, but current evidence is not sufficient to support HHV-7 specific diagnosis or treatment
Qi et al.	HSV-1	Mechanistic *in vitro* study using MRC-5, Vero, and 293T cells, wild-type HSV-1, and an ICP22 condensate-defective mutant	ICP22 condensates reduce RNAPII Ser-2P occupancy at host promoters while maintaining its distribution on the viral genome, thereby suppressing host transcription and favoring viral transcription	Evidence is restricted to cell culture models, including experiments performed at high multiplicity of infection; no animal or clinical validation was conducted	ICP22 condensate formation and its interaction with the host transcriptional machinery represent potential, but still unvalidated, antiviral targets
Song et al.	HSV-1	Bibliometric analysis of 1,076 herpes simplex keratitis-related publications indexed in the Web of Science Core Collection from 1941 to 2024, using VOS viewer, CiteSpace, and R.	Herpes simplex keratitis research has primarily focused on stromal keratitis management and recurrence, with growing interest in ultraviolet A- based approaches	The analysis was restricted to publications indexed in a single database (WoSCC), and bibliometric trends reflect publication and citation patterns rather than direct evidence of biological mechanisms or therapeutic efficacy	Identifies potential therapeutic priorities for preventing herpes simplex keratitis recurrence and improving stromal keratitis management, with ultraviolet A-based therapy emerging as an approach requiring further clinical validation
Tadros and Godiwala	HSV-1	Case report of neonatal HSV infection complicated by hemophagocytic lymphohistiocytosis (HLH)	Despite reduced systemic inflammation and viral load, the patient did not survive	As a single case report, the findings have limited generalizability and cannot establish broader conclusions about neonatal HSV-associated HLH	The case illustrates the serious nature of neonatal HSV infection and the importance of early risk assessment and preventive intervention
Wang et al.	VZV reactivation in systemic lupus erythematosus	Systematic review and meta-analysis of 51 studies including 246,822 patients with SLE	Prevalence of herpes zoster was 12.3%, and the incidence was 22.0 cases per 1,000 person-years; several treatments and clinical factors were associated with increased risk	Very high heterogeneity, limited geographical representation, possible publication bias, and predominantly observational evidence restrict causal interpretation and generalizability	Findings may inform risk assessment, surveillance, treatment decisions, and prioritization of vaccination in patients with SLE

A first thematic focus concerns intracellular mechanisms that govern the viral replication cycle. Two studies in this Research Topic highlighted how herpesviruses manipulate host nucleocytoplasmic trafficking. Cao et al. described how viral shuttle proteins control RNA and protein transport from the nucleus to the cytoplasm, thereby affecting mRNA stability, virion assembly, and replication. This broad framework was complemented by the mechanistic study by Qi et al., who showed that HSV-1 ICP22 forms nuclear condensates that sequester RNA polymerase II (RNAPII Ser-2P) from host promoters, shifting transcription toward the viral genome. While the review by Cao et al. integrated observations across multiple herpesviruses and proteins but provides no new experimental validation, the article by Qi et al. offered direct mechanistic evidence in the experimental systems examined, although the generalizability of this mechanism across cell types, viral strains, and *in vivo* infection remains to be established.

Another study in this Research Topic demonstrated that pseudorabies virus (PRV), a neurotropic herpesvirus, induces extensive metabolic reprogramming in infected porcine cells (Chen et al.). Metabolomic and transcriptomic analyses identified alterations in nucleotide metabolism, amino acid pathways, and biosynthetic processes during viral infection, highlighting nucleotide metabolism as a potential area for further investigation as an antiviral target. However, these omics data identified an association with viral replication rather than demonstrating a therapeutically exploitable dependency. Functional perturbation, metabolic rescue experiments, selectivity studies, and *in vivo* validation will therefore be required.

In addition to intracellular mechanisms, a second key focus concerns the balance between antiviral protection and immune-mediated pathology. The studies included in this Research Topic highlight the crucial role of innate immunity, particularly interferon signaling pathways, in determining the outcome of infection. Type I and III interferons represent the first line of defense against viral infections and play a crucial role in containing viral replication and activating adaptive immunity (Schneider et al., 2014; Ivashkiv and Donlin, 2014). In this context, one study in this Research Topic showed that patients with HSV-2 meningitis exhibit increased spontaneous type I and III interferon production and enhanced IFN-γ responses to viral stimulation (Bjerhem et al.). This phenotype is compatible with innate immune priming; however, because persistent epigenetic or metabolic reprogramming and enhanced responses to heterologous stimuli were not assessed, the study does not establish trained immunity. Clinical studies suggest that herpesvirus disease manifestations are associated with differences in host immune status and inflammatory responses; however, the nature and direction of these associations vary across the conditions examined, and the observational design of these studies precludes conclusions about causality. Bjerhem et al. found that stronger pro-inflammatory responses were associated with systemic or neurological manifestations (e.g., meningitis), whereas weaker responses correlated with localized genital disease, raising the open question of whether this reflects a pre-existing host predisposition or a consequence of the infection itself. In a different clinical context, Wang et al. identified lymphopenia, organ involvement, and immunosuppressive treatment as factors associated with an increased risk of herpes zoster in patients with systemic lupus erythematosus. Taken together, these associations are clinically informative but should not be interpreted as independent causal determinants of severity.

At the opposite end of the evidence spectrum, a case report included in this Research Topic demonstrated the consequences of hyperinflammation on disease status (Tadros and Godiwala). Neonatal herpes simplex virus infection can cause fatal disseminated disease and, rarely, trigger hemophagocytic lymphohistiocytosis (HLH), a hyperinflammatory disease. This case report highlights the challenges of managing concurrent HSV infection and HLH in a neonate. Despite reduced inflammation and viral load with treatment, the infant developed multiorgan failure, underscoring the importance of early diagnosis, prevention, and risk assessment. Collectively, disruption of immune homeostasis—whether through impaired antiviral control or excessive inflammatory responses—appears to promote viral reactivation and more severe disease across these heterogeneous clinical settings (Cohen, 2013), supporting a model in which the outcome reflects a context-dependent balance between antiviral competence and inflammatory injury, while the direction of causality remains unresolved.

In a different study, Marek's disease virus (MDV), an alphaherpesvirus, was used as a recombinant vaccine vector expressing VP1 and VP2 proteins of the chicken infectious anemia virus (CIAV) (Ge et al.). The vaccine induced robust humoral and cellular immunity, significantly reduced viral replication and disease severity, and demonstrated the potential of herpesvirus-based vectors for controlling CIAV infection.

The application of advanced molecular technologies also offers new opportunities for the detection of previously unrecognized viral signatures and the expansion of the diagnostic spectrum of herpesvirus-associated diseases. In this context, herpesviruses in PCR-confirmed adenovirus-negative conjunctivitis were investigated using metagenomic sequencing followed by targeted PCR. This approach identified HHV-7 DNA in conjunctival samples from individuals recovering from conjunctivitis, whereas HSV-1, HSV-2, and HHV-6 were not detected (Hartwick et al.). Although a causal role for HHV-7 was not established, the study demonstrates the usefulness of combining unbiased sequencing with targeted molecular diagnostics to identify unexpected viral signatures. Further studies are needed to clarify the clinical relevance of HHV-7 detection in conjunctivitis.

Beyond these specific findings, this Research Topic provides a broader view of the changing landscape of herpesvirus research. Through a bibliometric analysis of publications from 1941 to 2024, Song et al. identified stromal keratitis management and disease recurrence as major research areas, alongside a growing interest in emerging therapeutic strategies, particularly ultraviolet A therapy. However, bibliometric prominence reflects research activity and should not be interpreted as evidence of therapeutic efficacy.

Taken together, the studies summarized in this Research Topic contribute to our understanding of the complex interactions between herpesviruses and their hosts. Viruses have evolved sophisticated strategies to evade immune recognition, disrupt interferon signaling, and alter cellular homeostasis, whereas host responses, although essential for infection control, may also contribute to tissue damage and disease. These contributions collectively support three broader insights. First, herpesvirus-induced host remodeling operates across interconnected spatial, transcriptional, and metabolic levels. Second, disease outcome cannot be explained simply by the strength of the antiviral response, as it appears to depend on timing, anatomical context, and regulation. Third, high-dimensional technologies provide powerful hypothesis-generating tools, but their translational value depends on subsequent causal validation. Priorities emerging from this Research Topic include validating trafficking, condensate, and metabolic mechanisms in primary cells, organoids, and *in vivo* models; using longitudinal immune profiling to distinguish host predisposition from consequences of infection; defining rigorous criteria for assigning causality to unexpected viral detections; and testing candidate metabolic vulnerabilities through functional perturbation, rescue, toxicity, and pharmacological studies.
